# Occupational Therapists as Social Prescribers: Insights from Swedish Primary Care

**DOI:** 10.1007/s44474-026-00002-6

**Published:** 2026-07-16

**Authors:** Emilia W.E. Viklund, Frida Degerstedt, Frida Jonsson, Anna Sofia Lundgren, Ingeborg Nilsson

**Affiliations:** 1 Department of Community Medicine and Rehabilitation, Umeå University, Umeå, Sweden; 2 Department of Social Work, Umeå University, Umeå, Sweden; 3 Department of Culture and Media Studies, Umeå University, Umeå, Sweden

**Keywords:** Healthcare provider, Interviews, Loneliness, Older adults, Social activities, Wellbeing

## Abstract

**Background:**

Loneliness is increasingly recognized as a public health issue, with social prescribing gaining global interest as a useful approach to address social needs by connecting patients to non-medical community resources. Occupational therapists have previously been recognized as well suited for social prescribing initiatives, yet their role remains underexplored.

**Aim:**

This study aims to explore occupational therapists’ experiences of a social prescribing model as well as the professions’ role in applying it in Swedish primary care to alleviate loneliness among older adults.

**Material and Methods:**

Semi-structured individual interviews with 10 occupational therapists were conducted digitally. The data analysis was guided by reflexive thematic analysis.

**Results:**

The social prescribing model was perceived as potentially valuable to address loneliness among older adults in primary care. While occupational therapists felt that their professional expertise positioned them well to contribute to the approach, they also identified important constraints that limited their capacity in their role as social prescribers.

**Conclusion/Significance:**

To realize the social prescribing model’s potential in Swedish primary care, the complexity of loneliness and social activities must be acknowledged. Efforts are also needed to lower thresholds for social participation, and fostering collaboration across organizational and professional boundaries.

**Supplementary Information:**

The online version contains supplementary material available at 10.1007/s44474-026-00002-6.

## Introduction

Defined as the perception that one’s current social life does not meet one’s social needs [1], loneliness is a global health concern affecting one in six individuals worldwide [2], with significant health consequences [3, 4]. Yet loneliness has received limited attention in occupational therapy-related research [5]. It is also largely absent from influential theories and clinical practice, despite its links to familiar concepts such as occupational imbalance and deprivation [6].

While loneliness can occur across the life course, older adults have been seen as especially at risk due, for example, to loss of important others and changes in health status [7]. This prompted policy concern and initiatives for combating it both internationally and in Sweden [2, 8]. Specifically, a variety of interventions have been developed to alleviate loneliness, some of which have potential, such as group-based treatment, internet training, animal therapy, and group exercises [9, 10]. However, due to the subjective nature of loneliness and the diversity among older adults, tailored support for alleviating it is essential [11, 12].

By connecting clients, often in primary care, to non-medical community resources in line with individual’s needs, social prescribing is an initiative with the potential to reduce loneliness—and enhance wellbeing among older adults [13–15], prompting increased global interest during the last decade [16, 17]. Rather than being a standardized or manual-based intervention, social prescribing can take various forms, varying from simple signposting by healthcare personnel to more intensive support provided by “link workers” who help create a personalized plan that can include various social activities and services [16, 18]. Link workers are suggested to hold a key position in social prescribing, and the role requires good knowledge of the community’s social fabric, a person-centered and collaborative approach in client engagement, as well as a holistic view of health [16, 19]. The role can be a dedicated position, which is common in the UK, but it can also be carried out as an extension of existing care providers’ responsibilities, such as nurses, social workers, health promoters, or occupational therapists [16].

Occupational therapists’ role in social prescribing is largely underexplored, with only one study to date highlighting opportunities but also challenges in positioning the profession within this approach [20]. In this study, the similarities between occupational therapy and social prescribing were emphasized, noting that link workers carry out assignments that resemble occupational therapy, albeit often without having this training, which might threaten professional identity. Nonetheless, meaningful activity and social connectedness are seen as central to health within occupational therapy [21], and social prescribing may present an opportunity for occupational therapists to advocate for these principles to promote wellbeing among clients [20, 22].

In Sweden, Social Prescribing in Sweden (SPiS) has pioneered the testing of social prescribing in primary care [23]. Based on an ecological-transactional system approach, the Person-Environment-Occupation-Performance (PEOP) model [24] combined with literature on social prescribing initiatives from the UK, we developed a social prescribing model together with healthcare providers, which has been tested across primary care centers in Sweden. At many of the centers involved in the project, occupational therapists embraced the link worker role—referred to as social prescribers in the Swedish model—assessing needs and matching older adults with suitable and available social activities as an extension of their daily work. This study aims to advance the understandings of the potential role of occupational therapists in social prescribing. Specifically, it explores occupational therapists’ experiences of a social prescribing model as well as the professions’ role in applying it in Swedish primary care to alleviate loneliness among older adults.

## Material and Methods

### Study Design

A qualitative study design was used to capture the experiences of occupational therapists involved in the SPiS project. Semi-structured interviews were conducted, and an inductive thematic analysis approach was applied to data analysis. The framework for reporting qualitative studies (COREQ) [25] was followed during the drafting of the article and a completed checklist is included as supplementary material.

### Setting, Informants, and Recruitment

#### Swedish Primary Care

Sweden has a decentralized governance structure comprising the national government in addition to 21 regional councils (henceforth “regions”) and 290 municipalities. Following the principle of local self-government, the regions and municipalities have a considerable degree of autonomy and independent powers of taxation. The regions are responsible for specialized secondary care but also for primary care. Primary care is responsible for basic health and medical services while serving as the first point of contact, addressing a wide range of personal care needs through primary care centers. Primary care centers can be either public or private where private ones working under agreements with regional councils offer healthcare to the same cost as the public centers. The workforce of primary care centers varies, but commonly consists of general practitioners, nurses, physiotherapists, occupational therapists, and psychologists.

The municipalities, meanwhile, are responsible for other health-related services such as elderly care, including assisted living facilities, mobility support, and home care services, but also for social activities and education. Additionally, civil society and private sector organizations are also active in the Swedish welfare system, contributing to prevention and health promotion by offering various activities and support.

#### The Social Prescribing in Sweden project

The present study is part of the research project Social Prescribing in Sweden, which aims to evaluate the feasibility of a social prescribing model delivered at Swedish primary care centers, focused on improving health and alleviating loneliness among older adults. The social prescribing model draws on existing structures and resources, supporting primary care providers in identifying and intervening in situations of loneliness among older adults (aged 65 years and older) by guiding them to ongoing social activities in their local community.

The social prescribing model was co-developed by our multidisciplinary research team at Umeå University together with local primary care actors and a municipality, starting in 2018. The model was inspired by literature on social prescribing initiatives from the UK but revised and adapted to the Swedish context through participatory approaches and an iterative process of ideas, tests, and refinements during regular meetings with primary care actors and in dialogue with representatives from municipalities [23].

In 2023, an extended feasibility phase was initiated. Ten primary care centers located in six municipalities in Sweden voluntarily participated in the project to test the social prescribing model in real-world settings. Presentations and media outreach about the project during the co-development phase sparked interest among municipal and primary care managers who contacted the research team to join the research. Each center received an introduction, a step-by-step guide, and materials for the social prescribing process. They also had access to regular follow-up sessions with the project team where questions related to the project and the model could be discussed.

#### The Social Prescribing Model

**Fig. 1:**
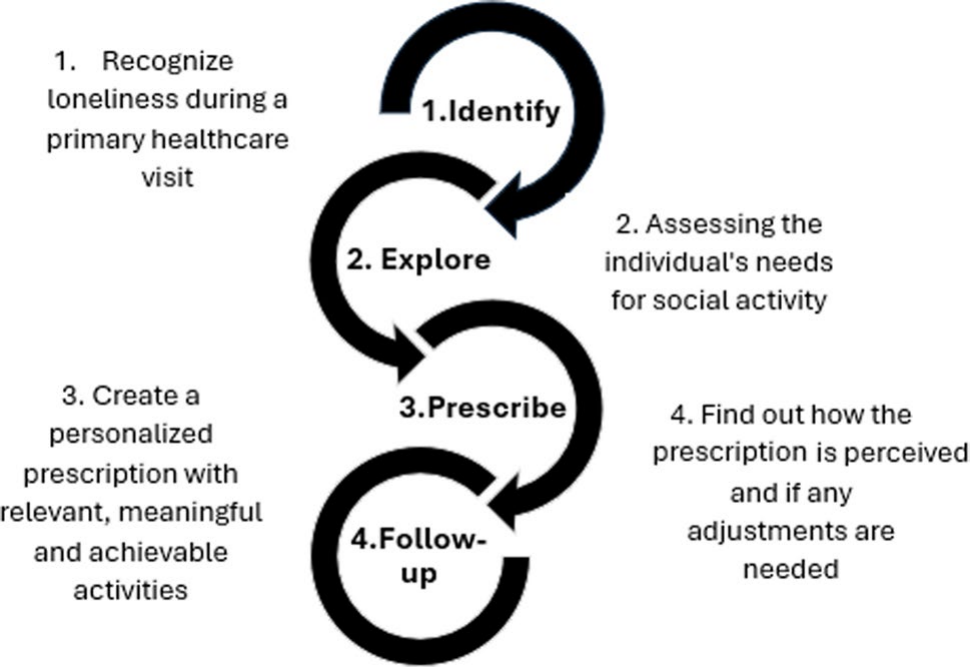
Illustration of the Swedish social prescribing model

The social prescribing model (in Swedish Social aktivitet på recept ) details how primary care providers should identify clients who experience loneliness, undertake an in-depth assessment of need for and interest in social activities, prescribe relevant and meaningful ongoing social activities in the community, and follow up on the prescriptions (see Fig. 1).

The first step of the model is to identify clients who experience loneliness by asking, “Are you troubled by loneliness?” All clinical staff at the participating primary care centers should ask this standardized screening question in meetings with clients aged 65 years and over as part of their routines. If the client confirms experiences of loneliness and expresses interest in trying social prescribing, the primary care provider should inform about the project. Thereafter, if the client wants to participate, the primary care provider refers internally to a “social prescriber” at the center for an individual assessment. The primary care centers can decide who function as social prescribers among the available registered healthcare providers (e.g., occupational therapists, nurses, psychologists). The social prescribing process (steps 2–4) was encouraged to be carried out by the same social prescriber.

The individual assessment constitutes the second step of the model and explores client’s daily life, needs, and interests related to social activities, supported by a guide. In addition to interests, the guide also includes questions on the preferable form of the social activity (e.g., small or large groups and live or online meetings), when (e.g., daytime or evening) and how often, the value of the activity (e.g., helping others or of interest to the person themselves), and if there are certain activities the person would like to avoid and other preferences (e.g., regarding potential costs or geographical areas). Based on the information gathered, the social prescriber and the client develop an activity profile.

In the third step, prescriptions containing recommendations of social activities are created in close dialogue with the client based on the activity profile developed during the individual assessment. The goal is to find a suitable match among ongoing local social activities which client and prescriber agree would be good to participate in. Each participating center was free to decide on strategies for obtaining knowledge about ongoing activities in the local community. The model further encouraged prescribers to write physical prescriptions on paper that the client could take with them, using a template containing information regarding the social activity of choice, how often and for how long the client should participate, the goal of participation, and dates for follow-ups. The prescription could be written in a separate session or in conjunction with the individual assessment, dependent on the amount of time available to social prescribers.

The fourth and final step of the model is the follow-up. The model suggests that a follow-up over telephone is carried out 3 weeks after receiving the prescription, following a guide aiming to explore if the client has participated in the prescribed activity and if any adjustments to the prescription are needed to better match individual needs. The model also includes a second follow-up, carried out face-to-face after 3 months, exploring the experiences of the prescription and offering an additional opportunity for adjustments.

#### Informants and Recruitment

This study focused on the experiences of occupational therapists participating in the Social Prescribing in Sweden project. At the primary care centers, various registered health care providers were involved but many of the participating centers choose to have occupational therapists acting as social prescribers.

In spring 2024, all occupational therapists involved in the project were invited to participate in semi-structured interviews. Invitations, including study information, were sent via email. One occupational therapist declined, resulting in ten informants who voluntarily gave their informed consent to participate.

The occupational therapists were all female with varied work experiences, ranging from 2 to 14 years in primary care (see Table 1). They represented different primary care centers in both urban and rural contexts across Sweden. As primary care centers joined the project at different times, the informants’ experiences with social prescribing varied in terms of time and number of prescriptions made.

**Table 1:** Background information on study informants

Background information	Total number of informants
Geographical work context	
*Location in Sweden*	
North Central South	3 5 2
*Setting*	
Urban Rural	8 2
Professional experience	
*Total experience as an OT (year)*	
5–10 11–15 20 +	4 4 2
*Experience in current primary care role (year)*	
2–5 6–10 10 +	4 5 1

### Data Collection

The study is based on ten semi-structured digital interviews conducted between April and August 2024. The interview guide included open-ended questions focusing on informants’ experiences of the social prescribing model, particularly exploring the model from their experiences and expertise as occupational therapists. It also focused on their views on occupational therapists’ role in applying it in Swedish primary care. The guide included questions such as the following: “Have you previously worked with or delivered interventions addressing loneliness?”, “How do you feel about taking on the role as a social prescriber as an occupational therapist in primary care?”, and “What would you identify as the main challenge in getting this model to function in practice?” The interviews, conducted by the last author who has extensive experience with interview-based research, lasted about 30 min and were audio-recorded and transcribed verbatim (50 pages in total). The first interview served as a pilot test of the interview guide, but as it was found to work well in its entirety, it was included in the study.

After generating preliminary results, all informants were contacted. Those interested were invited to share their thoughts on the preliminary results in one-on-one online meetings in December 2024 with the first and last authors. Five occupational therapists participated in these discussions, which were recorded and transcribed. Both the semi-structured interviews and online discussions were used for analysis.

### Data Analysis

Reflexive thematic analysis, as described by Braun and Clarke [26], guided the inductive data analysis. This flexible method emphasizes the active role of researchers in the dynamic and reflexive analysis process which began with data familiarization, including verbatim transcription and multiple readings of transcripts. The first author made the initial coding, by marking text sections or sentences regarded as relevant to the study aim and labeling these with key words. The coding was semantic, aiming to present the study informants’ perceived opportunities and challenges with the social prescribing model close to the raw data. These labels constituted the codes which then became the basis for the next step in analysis, grouping codes that reflected similar content. The codes were grouped, and re-grouped, several times and drafts of potential sub-themes and themes were shared, reviewed, and discussed in the author group. When the authors agreed upon a tentative structure and key findings, the first preliminary results were shared with informants for member checking [27], a technique enhances the credibility of the findings and aligns with the constructivist epistemology. The informants’ feedback guided the final part of the analysis. Discussing and reflecting upon the results in the author group and with the informants guided the analysis and constituted a core of the analytical process. The results were written concurrently with the analysis. The quotations included to illustrate the findings were translated by the first author with support of online translation tools and then checked by the other authors. They were translated after the data analysis, in conjunction with writing the manuscript. The findings include quotations from the interviews, followed by a code where “I” stand for informant and the next letter (A–J) indicates the specific interview.

### Ethical Considerations

The study was approved by the Swedish Ethical Review Authority (Dnr 2020-00659) and was conducted in accordance with the principles of the Helsinki Declaration [28]. Participation in the study was voluntary, and the informants could withdraw their consent to participate in the research at any time. The informants gave their informed consent to study participation and audio recording prior to initiating the interviews. The interview recordings and related personal information were handled in line with research integrity principles and university research data protection requirements, which operate in compliance with the European Union General Data Protection Regulation (2016/679). All raw data (interview recordings and unanonymized transcripts) were stored on secure university-owned servers fulfilling necessary data protection and data security requirements.

## Results

The reflexive thematic analysis resulted in the development of three themes and seven subthemes (see Table 2).

### “It is a part of our basics, actually”: Assessing Needs for and Interest in Social Activities

**Table 2:** An overview of the themes and sub-themes

Themes	Sub-themes
“It is a part of our basics, actually”: Assessing needs for and interest in social activities	Making a previously implicit focus explicit Trained in tailoring activity-related prescriptions
“It’s hard to keep track and be the spider in the web”: Connecting older adults with meaningful social activities	Balancing tailored options with practical limitations Managing concerns about letting lonely older adults down Finding the time to focus on non-urgent matters
“It’s not a quick fix”: Enabling participation in social activities	Lowering the thresholds for moving from prescriptions to participation Importance of noting the limits and barriers

The first theme highlights the importance of tailored prescriptions and how the expertise of occupational therapists can contribute to making multidimensional assessments exploring older adults’ needs, interest, and prerequisites.

#### Making a Previously Implicit Focus Explicit

Consistent with the social prescribing model, informants agreed on the significance of crafting tailored prescriptions in line with individual needs and interests. Across the interviews, informants detailed how the focus of the model—loneliness and social activities—are well connected with fundamental values of occupational therapy, namely activity, participation, and meaningfulness. As informant D noted:

“For me, it has felt so obvious that this [social prescribing] is occupational therapy.” (ID).

The informants noted how the initial step of the model, assessing individual needs, closely resembled their usual work as occupational therapists—though with a sharper focus on social activities. While the social life of clients was often touched upon in client interactions even before their involvement in the project, these aspects had rarely been at the center or acted upon. Instead, these aspects tended to remain in the background, as one informant described:

“I think it’s a part of our basics, actually. […] But we haven’t really worked with it actively before.” (IA).

#### Trained in Tailoring Activity-Related Prescriptions

Within this context, the research project and the social prescribing model were a welcomed addition to address loneliness, a concern that informants had previously felt uncertain about tackling. Overall, occupational therapists were considered well-suited for tailoring prescriptions of social activities to older adults experiencing loneliness. Their expertise and training in conducting assessments to identify individual needs were perceived as highly relevant and they related strongly with the idea that older adults’ own views of what feels interesting and meaningful must guide the prescriptions. This, they noted, requires sensitivity, a skill that was seen to develop with experience and as one informant reflected, involves holding back one’s own professional or personal views. Informants highlighted the power imbalance between social prescribers and clients, and that some older clients otherwise might agree to suggestions without expressing their true preferences.

Besides exploring social activities that were interesting to clients, informants emphasized the importance of assessing their broader needs, symptoms, and life situations, noting that loneliness might co-occur with various health problems in old age, and that social prescribing might not be suitable for everyone. They considered occupational therapists’ medical expertise and knowledge of functional limitations, combined with a multidimensional view of activities—carefully considering the activity, person, and the environment—as central in the individual assessments. In fact, they saw it as unique, as something that distinguishes occupational therapists from other health professions as described by one informant:

I think we are able to contribute based on our holistic view on things. And precisely this aspect of activity, person and environment. […] It is easy for someone else to suggest that you can play pétanque, but that may not work if you take all aspects into consideration. (IC).

Taken together, this first theme describes perceptions that the social prescribing model’s focus on meaningful social activities aligned well with occupational therapy, and the informants felt that their expertise and experience in making assessments could support the creation of tailored social prescriptions.

### “It’s hard to keep track and be the spider in the web”: Connecting Older Adults with Meaningful Social Activities

The second theme details challenges but also strategies for matching individual assessments with the available local supply of social activities within the professional remit as primary care occupational therapists.

#### Balancing Tailored Options with Practical Limitations

Matching individual needs with available local activities was described as a major challenge. Informants described that information on social activities was hard to find, often due to poorly updated websites and contact information, especially those run by associations. One informant likened the task to “detective work,” requiring time and resources that were scarce in primary care, where shortage of personnel, efficiency norms, and competing priorities shaped their practices. Informant I highlighted that under those circumstances, it was difficult to allocate the time necessary to acquire an overview of available options:

“I think that it is about finding these social activities. […] As occupational therapists, we can’t really… In our work, it’s difficult to keep track of everything and be the spider in the web this way. And I think it’s a great and significant part of making this [social prescribing] work.” (II).

Because of the perceived difficulties with locating social activities, informants suggested that any large-scale implementation of social prescribing would benefit from a designated person, a coordinator, responsible for gathering and keeping track of social activity information. This, they argued, would facilitate the process and allow occupational therapists to concentrate on the area where they perceived they could contribute most—the individual assessments. Yet some informants had found strategies for managing the challenges by collaborating with municipalities and civil society to get support with compiling information in continuously updated folders at the primary care center.

While some informants struggled with acquiring an overview of available social activities, others had problems finding activities at all. Challenges related to having too few options were especially highlighted by informants in rural areas, where the limited supply was experienced to put informants in a difficult position, making them hesitant to even initiate the social prescribing process. Informants described how sometimes quite detailed interests and requirements among some older adults were difficult to meet with available local resources. Some had also encountered misunderstandings of the social prescribing process, where older adults had expected primary care to create new activities specifically for them, rather than matching with ongoing options*.* Nevertheless, one informant reflected that expectations and pressures related to delivering a prescription that was a “perfect match” with their interests were perhaps more intrinsic, driven by informants own desire to perform their job well, than being expressed by older clients. One informant described worrying about raising false hopes in her clients by offering social prescribing, reflecting on the risk that her inability to deliver prescriptions of individually meaningful activities could make older clients:

“feel even more lonely after they have met me. […] That’s how it is. You are afraid of that.” (IF).

Similar concerns about social prescribing, that rather than alleviating loneliness and promoting wellbeing, it would worsen the situation of older adults were also expressed by another informant. She specifically cautioned that:

In a way, you’re recommending something that could also happen to be. Yes. Bad. […] there could be contacts that aren’t good for the person. (IJ).

#### Managing Concerns About Letting Lonely Older Adults Down

The importance of adopting a mindful approach was emphasized, noting that prescribing unreliable social activities could unintentionally legitimize them and put already vulnerable people at risk.

Concerns about disappointing older clients were also frequently raised. However, while most informants experienced the process of connecting older adults with relevant social activities as stressful, some also seemed to have taken a more relaxed approach. Informant J shared how, instead of attempting to search for suitable activities independently, she had progressively adopted a more collaborative approach, involving the clients in the search process. By seeing the prescriber role as more of a facilitator than an expert, the pressures of finding a “perfect match” were revealed:

But it’s like a process with the client. That you get to brainstorm together and figure things out. […] I don’t need to be up to date on what’s happening everywhere. Instead, we sit down at the computer and see what’s available. (IJ).

#### Finding the Time to Focus on Non-urgent Matters

The current organizational landscape within primary care was also perceived to challenge the implementation of the social prescribing model. Informants described that their work was highly influenced by a lack of resources, which was seen as affecting their opportunities to engage with the approach. They highlighted feeling that a shortage of personnel led to having to prioritize “more urgent matters” with social prescribing not being regarded as such. Primary care was further seen as focused on efficiency and informant E described feeling pressured to produce “good statistics,” which was seen as leading to difficulties in making social prescribing a priority:

Unfortunately, I don’t place it [social prescribing] on top the list. I don’t think so. […] When it comes to spending time there and prioritizing other things, it’s not measurable in the same way as if I were to treat eight carpal tunnel cases in one day. Then I would quickly get statistics on how they did. (IE).

In sum, the second theme highlights challenges with connecting lonely older adults with relevant social activities in the local community, which made informants question whether they could or should take on the role of prescribers. However, while struggling with limited resources and emotional concerns about letting older persons down by failing to find meaningful options in the scattered and sometimes scarce supply of local social activities, informants had also found ways to manage the constraints. By partnering up with activity providers and older adults themselves the gaps could potentially be bridged.

### “It’s not a quick fix”: Enabling Participation in Social Activities

The third theme captures a perceived need to expand the prescriptions to also include support for enabling participation to make a difference for as many older adults experiencing loneliness as possible but also highlights the importance of keeping it manageable.

#### Lowering the Thresholds for Moving from Prescriptions to Participation

The complexity of loneliness was frequently mentioned, with informants noting that supporting older adults to find meaningful activities alone may not be sufficient to alleviate it. Beyond tailoring prescriptions to individual interests, needs, and available social activities, they emphasized that some older adults might require additional support to put prescriptions into practice. One informant described an example of an older woman who enjoyed dancing, noting that a prescription for dancing only becomes meaningful when the barriers are not only acknowledged but also addressed, as she put it:

It is about enabling so that [the client] is able to dance, which she enjoys. But she has various difficulties that make it not entirely possible. Then that should be included in the prescription as well. That is when it becomes meaningful. (IG).

Informants highlighted that the prescription needs to not merely include suggestions of meaningful social activities but also strategies to enable engagement. They noted how various barriers may prevent older adults from engaging in social activities, and that the barriers remain even after receiving a prescription if they are not addressed simultaneously in the social prescribing process. As described by informant H:

“Because, of course, you can guide them to the right activity, but then they have to get there, maintain it and carry it out.” (IH).

One recurring form of support described by informants related to enabling access to social activities was with transportation. Informant E shared an example of an older client that knew which activities he wanted to participate in but experienced difficulties with moving around. In this case, an electric wheelchair was prescribed, allowing him to participate and access the activity. Beyond physical barriers, informants noted psychological obstacles, such as negative beliefs, anxiety, or social phobia, which made some older adults feel nervous about participating, especially in group activities. It was emphasized that many older adults may need emotional support to take the first step and fostering motivation was frequently described as playing a crucial part of the prescriber role. Offering such support was perceived to align with occupational therapists’ skills in supporting processes of “practicing, rehearsing, and trying out. […] To finally be able to do” (IA), as described by informant A. However, informants also noted, this may require additional resources and sessions that are added to the social prescribing model.

It was further highlighted by informants that some barriers to realizing prescriptions can be addressed by occupational therapists, while others require support from other professionals and organizations. Collaborating with activity leaders to ensure a welcoming environment, especially during initial visits, was seen as crucial for addressing psychological barriers. Additionally, having a companion accompany the individual to and from the activity was seen as a potential facilitator. While some noted a lack of such services in their communities, others had successfully arranged with municipalities to provide companions for initial visits. Informant B further pointed out that older adults’ own informal networks can serve as a resource for supporting participation in social activities:

It’s really about looking around. What kind of networks do they have? Do you have someone — a relative, for instance — who can maybe drive you there? A neighbor, perhaps, or someone else nearby who’s going to the same place and then check whether it would be okay for you to go together. (IB).

#### Importance of Noting the Limits and Borders

While informants noted the need to, in many cases, include support to address potential barriers in guiding older persons toward suitable activities, they also cautioned against taking on more than one can manage. Loneliness was described as “a big deep pit,” highlighting its complexity and the importance of staying within one’s competencies and the scope of the model. Informant A described the need to set clear boundaries to be able to make the prescriber role in primary care manageable:

It’s really not a quick fix. So it’s about finding some kind of boundary. What can I do, what do you need to work on yourself, and what do you need help from others for? Otherwise, you can get swallowed. (IA).

Taken together, this theme emphasized the importance of making sure that the social prescribing model actually works in everyday practice—both from the prescribers’ perspective and for the prescriptions to genuinely make a difference for the older adults. Addressing potential barriers for participating in social activities was seen as key for making the prescriptions matter and informants emphasized that strategies and support for lowering thresholds should be included in the prescriptions and the social prescribing process. However, they also emphasized the need to see the model for what it currently is and avoid making its focus too broad, as this could render the social prescriber role unmanageable within the current state of primary care.

## Discussion

This study aimed to contribute to the literature by exploring occupational therapists’ experiences of a social prescribing model to address loneliness among older adults, as well as their perspectives on the profession’s role in applying such model in everyday practice in Swedish primary care. While the findings suggest that occupational therapists are, in many ways, well positioned to contribute meaningfully to social prescribing, the study also highlights substantial challenges with applying the model into daily practice with the current resources available.

One of the most notable differences between the Swedish model and other approaches to social prescribing, such as in England, is that the social prescribing process is carried out by registered primary care providers rather than by newly introduced roles such as link workers [29]. Consequently, these tasks are supposed to be integrated into their existing professional responsibilities. In many cases in this Swedish project, this role fell to occupational therapists, who then took on the task of assessing social needs, identifying available social activities, and matching the two in a social prescription and following up on them.

The findings indicate that occupational therapists hold certain competencies that position the profession particularly well in the role as social prescribers. The social prescribing model was generally viewed as well aligned with occupational therapy values and practices, a finding that mirrors the experiences reported by occupational therapists in a study by Bradley et al*.* [20]. The occupational therapists’ expertise in activity-based assessments and their contextual understanding was seen by informants in our study as enabling them to tailor social prescriptions to individual circumstances—an element that has been identified as critical for making social prescribing [30, 31] and loneliness interventions work [11, 12]. Additionally, the findings also highlight that social prescribing might strengthen occupational therapy by offering an opportunity to more explicitly focus on core professional values related to meaningful activities and social connectedness [32]. Gallagher and Bagatell [33] argue that the dominance of biomedical perspectives and market-driven models of healthcare has distanced practice from these foundational values. Echoing previous studies [5, 6, 34–36], the results also suggest that social dimensions of health continue to receive limited attention in occupational therapy practice despite being regarded as important.

This study also highlights challenges in applying a social prescribing approach into daily occupational therapy practice in primary care. Challenges related to identifying suitable social activities were particularly evident, and when informants felt unable to offer prescriptions aligned with older clients’ needs and preferences, this generated stress (*cf* [18, 37]). Developing an overview of a fragmented and dynamic social activity landscape required substantial time that informants felt was unavailable given competing work tasks, lack of clear mandate to prioritize this work, and the overall pressure within primary care. However, most informants had limited experience with the social prescribing model when interviewed and such contextual knowledge may develop over time [37]. Indeed, some informants had found facilitating strategies, such as collaborating with activity providers and adopting a participatory approach involving older adults in identifying relevant activities.

Additionally, the findings also captured perceptions of a scarce supply of available social activities, which further complicated the prescribing process. These experiences were reported by some of the informants working in rural areas, aligning with experiences reported by social prescribers in Canada [38]. While online social activities could be an option for mitigating geographical differences, they received limited attention in the current study, potentially reflecting a lack of online social activities, limited awareness of them among informants, and/or prevailing stereotypical beliefs about older adults’ digital (in)competencies [39].

The findings further highlight the importance of considering the complexity of loneliness and that many older adults may need support extending beyond identifying social activities for reducing loneliness, which correspond with experiences presented by social prescribers and lonely older adults elsewhere [40–42]. With a professional remit extending beyond social prescribing, occupational therapists can provide additional support, such as motivational support or prescribing assistive devices, to enable older adults to participate in social activities. However, collaboration with municipal social services and activity providers was also seen as a key factor for addressing potential barriers, reflecting the study results of Gorenberg et al*.* [31]. Particularly, arranging transportation services and companionship were seen as important and the older adults’ own social networks were highlighted as potential assets for solving practical problems.

Although informants perceived that the social prescribing model may need to be expanded to address potential barriers more explicitly while identifying suitable activities, they also noted the risk of the social prescriber role becoming too broad and difficult to manage. Previous studies describe perceived challenges among link workers in encountering complex and intersecting needs among patients referred to them [18, 43, 44], and they emphasize the importance of clearly communicating the boundaries of the role early on [37]. However, this becomes more complex when social prescribing is embedded within an existing clinical profession. When occupational therapists act simultaneously in both roles, clients may expect a broader range of support, and practitioners themselves may find it challenging to separate responsibilities tied to each role.

The findings of this study draw further attention to the importance of being aware of the complexity of social connections and activities, and that they may not always be beneficial. In line with what Twinley et al*.* [45] refers to as “the dark side of occupation,” informants noted risks with social activities, potentially worsening the situation of already vulnerable persons. While such concerns are valid considering literature on the negative aspects of relationships and social activities [46] and evidence indicating loneliness as a risk factor of fraud [47, 48], these expressions may also be seen to reflect a paternalistic view. On the one hand, informants emphasized the need to respect and support older adults’ autonomy in defining meaningful activities. On the other hand, they expressed feeling responsible for ensuring that these activities were safe and protecting lonely older adults from potential disappointments. This ambivalence in how older adults were discussed reflects what Timonen [49] describes as the paradox of (in)dependence in active ageing discourses, where older adults are often simultaneously framed as both resourceful and vulnerable. While the potential dark side of social prescribing needs further exploration and should be recognized, overprotection may undermine a person-centered approach, central to social prescribing [29] and occupational therapy [50].

### Methodological Considerations

It is important to acknowledge the central role of researchers throughout the study. Research is shaped by its time, context, and the researchers themselves [26]. While being a necessity for data analysis, researchers’ preunderstanding is also a potential source of bias, influencing everything from the interview guide to the interpretation of the study findings [26].

However, the interdisciplinary author team, with disciplinary backgrounds in occupational therapy, physiotherapy, public health, ethnography, and developmental psychology, can be considered a study strength, potentially reducing the risk that individual preunderstandings dominate the analysis. Working across disciplines introduces multiple interpretive lenses and fosters reflexivity.

The fact that the preliminary findings were shared with the informants can be seen as an additional merit, strengthening the connection between the analysis [27] and the experiences and views of the informants while adding reflexivity.

It is also important to note the risks of social-desirability bias, as the informants, due to the power imbalance in the interview situation, may have hesitated to share more critical views. Additionally, the digital format may have limited interaction during the interviews. On the one hand, digital interviews have been suggested to reduce the richness of interview data [51], but on the other hand, they also enabled participation of busy primary care occupational therapists.

The relatively small sample size can be considered another study limitation. A small sample size may limit the variation in experiences and perspectives captured, which in turn may reduce the breadth of the findings. Additionally, the fact that all informants were women may have limited a diversity of viewpoint which potentially could have enriched the analysis. However, the informants varied in terms of their overall professional experience, their experience of working in the primary care and the geographical regions in which they were employed, which may have contributed to different perspectives.

Nevertheless, the small sample size does not necessarily limit the transferability of the study findings. In line with Braun and Clarke’s [52] argument that meaningful qualitative insights do not depend on achieving saturation, and with Drisko et al*.*’s [53] view that transferability is grounded in the depth and contextual clarity of the data rather than statistical representativeness, even a small number of informants can offer analytically useful and transferable insights. Moreover, we identified parallels between our findings and those reported in studies conducted in other contexts, suggesting that the experiences described by our informants may resonate beyond the immediate study setting.

Finally, trustworthiness was promoted by detailed descriptions of informants, data, and procedures, while protecting informants’ integrity.

## Conclusion

This study highlights primary care occupational therapists’ experiences of a social prescribers’ model to alleviate loneliness among older adults as well as their perceptions of the profession’s role in applying it in Swedish primary care. While the findings suggest that occupational therapists are, in many ways, well positioned to contribute meaningfully to the social prescribing model, the study also highlights substantial challenges in integrating the model into daily practice with the current resources available. To realize the social prescribing model’s potential in Swedish primary care, the complexity of loneliness and social activities must be acknowledged. Efforts to reduce barriers to social participation and strengthen collaboration across organizations and professions were viewed as essential both for enabling social prescriptions to make a meaningful difference for lonely older adults and for ensuring that the model is manageable for social prescribers.

Author contributions

## Supplementary Information

Below is the link to the electronic supplementary material.

ESM 1 (23.9 KB DOCX)

## Data Availability

The dataset underlying this article is not publicly available but can be shared on reasonable request from the corresponding author.
