# Supplementary material for: Occupational Therapists as Social Prescribers: Insights from Swedish Primary Care
Source: Scand J Occup Ther. 2026 Jul 16;33(1):2. doi: 10.1007/s44474-026-00002-6 (PMC13385134; doi:10.1007/s44474-026-00002-6)
Supplement: Supplementary file 1 — (23.9 KB DOCX) [file 44474_2026_2_MOESM1_ESM.docx]

COREQ 32-item checklist for interviews and focus groups (Tong, Sainsbury & Craig, 2007).

| **Research team and reflexivity** |  |  |
| --- | --- | --- |
|  | Interviewer/facilitator | The last author conducted all of the interviews. The first and the last author facilitated the discussions on the preliminary results. |
|  | Credentials | First author = PhD  Second author = PhD  Third author =PhD  Fourth author = PhD  Fifth author = PhD |
|  | Occupation | First author = Postdoctoral researcher  Second author = Postdoctoral researcher, physiotherapist  Third author = Associate Professor  Fourth author = Professor  Fifth author = Professor, occupational therapist |
|  | Gender | All of the researchers are female. |
|  | Experience and training | All authors have experience conducting qualitative interview studies. The more senior researchers have extensive experience in planning and carrying out both national and international research projects using qualitative methodologies, as well as in the broader field of health sciences. |
| **Relationship with participants** |  |  |
|  | Relationship established | The researchers were not employed by any of the organizations that the practitioners represented. The recruitment of informants reports no bias grounded on dependency issues. |
|  | Participant knowledge of the interviewer | All of the informants knew which organizations the researchers represented and their research interests. The informants and the researchers did not know each other privately; they had only come into contact through the research project |
|  | Interviewer characteristics | The interview guide consisted of open-ended questions that allowed informants to speak freely about their experiences and reflections. The potential power imbalance between the interviewer and the informants is acknowledged as a possible source of bias in the methodological section of the discussion. |
| **Theoretical framework** |  |  |
|  | Methodological orientation and theory | Reflexive thematic analysis (Braun & Clarke, 2021) |
| **Participant selection** |  |  |
|  | Sampling | Purposeful sampling |
|  | Method of approach | All occupational therapists that participated in the larger research project were invited to the interviews. |
|  | Sample size | 10 |
|  | Non-participation | One of the eligible participants declined taking part of the interviews. |
|  | Setting of data collection | The interviews were all conducted online as part of a larger research project. |
|  | Presence of non-participants | - |
|  | Description of sample | All study participants were registered occupational therapists and women and their work experience varied between two to fourteen years from primary care. They worked at different primary care centers, representing various geographical locations in Sweden and both urban and more rural areas. |
| **Data collection** |  |  |
|  | Interview guide | A semi-structured interview guide encompassing broad themes and related questions guided the interviews. The first interview served as a pilot where the guide was tested. |
|  | Repeat interviews | - |
|  | Audio/visual recording | The interviews were audio recorded. |
|  | Field notes | - |
|  | Duration | The interviews lasted approximately 30 minutes |
|  | Data saturation | All individuals who met the inclusion criteria (being an occupational therapist involved in the research project) were invited to participate. In line with Braun and Clarke’s argument that meaningful qualitative insights do not depend on achieving saturation, even a small number of informants can offer analytically useful and transferable insights. |
|  | Transcripts returned | - |
| **Data analysis** |  |  |
|  | Number of data coders | The first author conducted the initial coding of the data and the first and last author generated the themes which were reviewed several times by the full author group. |
|  | Description of the coding tree | Table 2 displays the sub-themes and themes. |
|  | Derivation of themes | The themes were developed from the codes in an iterative and dynamic process of reading the transcripts, grouping codes based on similarities, forming and re-arranging groups of codes. |
|  | Software | Microsoft Word |
|  | Participant checking | Informants who volunteered received a preliminary version of the results which was later discussed with them in short one-to-one online discussions. |
| **Reporting** |  |  |
|  | Quotations presented | The reporting of the findings include quotations from the raw data file (translated from Swedish to English by the authors and with the support of online translation services). |
|  | Data and findings consistent | The data analysis and the results were discussed at several times within the author group. Additionally, the preliminary results were discussed with the informants. |
|  | Clarity of major themes | The themes are described in text as well as in Table 2. |
|  | Clarity of minor themes | The sub-themes are described in the text as well as in Table 2. |
